# Scale of Body Connection: A multi-sample construct validation study

**DOI:** 10.1371/journal.pone.0184757

**Published:** 2017-10-13

**Authors:** Cynthia J. Price, Elaine Adams Thompson, Sunny Chieh Cheng

**Affiliations:** 1 Department of Biobehavioral and Health Informatics, School of Nursing, University of Washington, Seattle, Washington, United States of America; 2 Department of Psychosocial and Community Health, School of Nursing, University of Washington, Seattle, Washington, United States of America; 3 Nursing and Healthcare Leadership Program, University of Washington, Tacoma, Washington, United States of America; Hospital Universitario de la Princesa, SPAIN

## Abstract

The *Scale of Body Connection* (SBC) was created to address the need for a self-report measure to examine body awareness and bodily dissociation in mind-body research. Developed in the U.S.A., it has been translated into many languages and tested for validity of scale translation. The burgeoning of mind-body research and the widespread use of the SBC scale underscored the need for critical assessment of the instrument’s measurement properties. Thus, a broader evaluation of the SBC was designed using large samples from eight international, cross-sectional studies drawn from community (i.e., non-clinical) populations. Specifically, we assessed scale distribution properties and internal consistency reliabity, and using confirmatory factory analysis we evaluated scale contruct validity and compared male/female measurement models. The results indicated acceptable reliability for both the body awareness and bodily dissociation scales, and a good fit between the proposed theoretic model and the data, providing evidence of construct validity across all samples. Mean differences in body awareness were observed for males vs. females in most samples, with females generally showing higher body awareness compared to males. Multi-group structural equation modeling demonstrated a stable latent factor structure and factor loadings, indicating equivalent measurement models for males and females. In summary, this multi-sample study demonstrated SBC construct validity that supports its use in clinical research as a brief, readily translated, easy to administer measure of body awareness and bodily dissociation.

## Introduction

There is considerable scientific interest in furthering the understanding of body awareness, the clinical benefits of intervention approaches that target the capacity for body or interoceptive awareness and practice, as well as understanding the neurological, behavioral, and physiological regulatory links to body awareness. With growing interest in interoception in cognitive neuroscience and related fields, particularly mindfulness transdisciplinary research, it is important to critically evaluate measures of body/interoceptive awareness to ensure standardized use of such measures in these expanding fields. A systematic review identified many of the initial measures developed and/or tested to examine body awareness.[1] Each of these measures, from one of the first[2] to one of the most recent,[3] is typically designed to address a specific research question. For example, The *Scale of Body Connection* (SBC) [4], was one of the first body awareness measures developed and tested expressly for mind-body intervention researchand is the only such measure that includes a scale specific to bodily dissociation. The SBC has been used in numerous published studies around the nationally and internationally—including both cross-sectional studies to examine the scale validity in another language [5–10], and to better understand the role of body connection in health, and intervention studies to examine change in body awareness and/or bodily dissociation [6, 11–20]. The majority of the SBC body awareness (vs. bodily dissociation) items were included in the Multidimensional Assessment of Interoceptive Awareness (MAIA)[21], a subsequent measure designed for mind-body research. Given the apparent need for such measures, continued validation is important for behavioral and neuro-physiological research. It is not often that a systematic psychometric evaluation of a published measure is possible. This first multi-sample construct validation study of a body awareness measure was achieved through the cooperation among the researchers involved (see acknowledgements).

Improved interoception has been suggested as the mechanism underlying mind-body and mindfulness-based treatment approaches for multiple conditions (e.g., chronic pain, depression, PTSD and substance use) [22]. Interoception is the sensory process of receiving, accessing and appraising internal bodily signals, and motivating behavior in the pursuit of desired physiological states [23]. Interoceptive *awareness* is the conscious ability to identify, access, and evaluate internal body sensations [24]. The majority of mindfulness-based and mind-body therapies are designed to develop an increased capacity for interoceptive awareness. Previously more narrowly defined [25], the broader definitions now cast interoceptive awareness as a multidimensional construct that takes into account how people attend to, appraise and respond to bodily sensations [26, 27]. Thus the constructs of body awareness and bodily dissociation are integral to our understanding of key interoceptive awareness processes [22]. Attention to the body and related regulatory habits matter because many body sensations are inherently valenced to motivate behavior, such as the pleasure of feeling relaxed or the aversion to a sudden pain or emotional trigger. Such ingrained, affective components to interoceptive signals, which most likely originally evolved to help humans maintain homeostasis [28], may serve to guide effective emotion regulation [29] and decision making [27].

The *Scale of Body Connection* (SBC) was developed to measure change in body awareness and association processes that underpin mind-body therapies. Initially published in 2007 [4], the SBC was tested with a sample of 291 male and female undergraduate students. Confirmatory factor analysis indicated acceptable goodness-of-fit indices and revealed Body Awareness (SBC-BA) and Bodily Dissociation (SBC-BD) scales as independent dimensions (*r* = -.08). *Body awareness* involves the ability to experience inner bodily sensations (e.g., restricted breathing, tension), identify links between physical sensation with emotion (e.g. shallow breathing and anxiety), and to “listen” to the body to guide self-care (e.g., need for rest, attend to emotions linked to a stressful event). Body awareness thus involves attending to bodily information in daily life, noticing bodily responses to emotions and/or environment. *Bodily dissociation* is a sense of separation from body, due to avoidance or emotional disconnection. Bodily dissociation is characterized by the avoidance or disregard of internal experience that interferes with health and self-care. Although linked to dissociative experiences and clinical diagnosis [30], bodily dissociation is not considered pathological [31]. The SBC instrument includes dimensions with items important for the study of interoceptive awareness [1]; it is also useful as an intervention process measure and is a viable indicator of intervention mediation mechanisms [31, 32].

Since its initial development and testing, the SBC has been translated into multiple languages and widely used in research. A critical review of the instrument’s construct validity is needed to ensure its structural integrity for global use with community samples, as the initial SBC construct validity was established using a relatively young student sample, not representative of the general population. In addition, the initial validation used the English language version of the scale, compelling this examination of potential differences in construct validity across cultures/languages. To date, other single sample validation studies of the translated scale have been completed, two of which have been published [5, 7]. This study using multiple samples was designed to describe the psychometric properties of the SBC scale, and to assess construct validity of the previously reported two-dimensional model using confirmatory factor analysis (CFA), including tests for measurement invariance between males vs. females.

## Methods

Research methods and procedures were reviewed and approved by the University of Washington Institutional Review Board (IRB), prior to initiating this research. To examine the structural integrity of the SBC, we requested datasets from individual investigators who had previously requested use of the SBC from the first author, and from other investigators who had reported use of the scale in publications. Investigators expressing interest in contributing their data were asked to provide: an anonymous dataset including all SBC scale items, demographic variables (e.g., sex, age, race/ethnicity, education, income), and a brief description the study design, target population, and research methods.

### Sample

The eight study samples were drawn from non-clinical community populations with broad age distributions, and from university student populations with more limited age distributions. The datasets are from studies conducted in multiple countries/cultures; five of these studies were designed to validate a translated version of the SBC scale (Table 1). All scale translations were conducted using translation and back-translation methods to ensure culturally appropriate meaning of scale items [5, 6]. For ease of referencing the study samples, we refer to the datasets by country, and in Table 1 we indicate the language of translation.

**Table 1:** Description of sample datasets.

Study	Country (translated language)	Type of Sample	Recruitment Process	Data Collection Mode	Sample Size	Age range (Median)	Female N (%)	Male N (%)
1	Italy (Italian)	Community	Covenience	Online	576	17–72 (27.0)	396 (68.8%)	180 (31.3%)
2	France (French)	Community	Convenience	Online	198	19–70 (39.0)	181(31.4%)	17 (8.6%)
3	Netherlands (Dutch) [6]	Undergraduates	Convenience	In-Person	434	16–38 (20.0)	321 (75.57%)	103 (24.29%)
4	Portugual (Portuguese) [5]	Community	Convenience	Online	909	18–72 (31.0)	445 (49.0%)	464 (51.0%)
5	USA (a) [4]	Undergraduates	Convenience	In-Person	291	16–46 (20.0)	162 (57.7%)	119 (42.3%)
6	USA (b)* [21]	Somatic Therapists	Purposive	Online	290	18–79 (48.5)	290 (100%)	–––
7	USA (c) [10]	Lesbian Women	Convenience	Online	328	21–79 (49.0)	240 (78.7%)	65 (21.3%)
8	Israel (Hebrew)	Undergraduates	Convenience	In-Person	608	18–54 (28.0)	377 (62.0%)	231 (38.0%)

*USA (b) sample included only females. Missing values indicated by dashes (–).

### Measures

The SBC [4] is a 20-item self-report measure with two distinct dimensions: body awareness and bodily dissociation. Twelve items measure body awareness (i.e., conscious attention to sensory cues indicating bodily state, for example, tension, nervousness, peacefulness). Eight items measure bodily dissociation (i.e., sense of separation from body, emotional disconnection). Item response options are based on a 5-point Likert-type scale measuring frequency of experience across a specific period. The researchers who provided datasets used in this study retained the initial SBC instructions, which specified the timeframe as the last two months.

### Data analysis

#### Distributional properties and scale reliability

Descriptive statistics (means, standard deviations, skew, kurtosis) were used to summarize the distributional properties of the SBC. Internal consistency reliability was assessed using Cronbach’s alpha. Analyses were conducted using SPSS, version 19.

#### Construct validity

Confirmatory factor analysis (CFA) was used to evaluate each dimension defined by the SBC theroretical measurement model, describing the factor structure and the relative size of item loadings [33] across samples, using multiple indicator CFA with M-plus 7.31 [34]. For each dimension, an initial CFA was conducted using the original English version of the SBC (USA a)[4] as a basis for CFA comparisons with the translated versions. Model fit indices included the Comparative Fit Index (CFI) (acceptable value ≥ .95), Tucker-Lewis Index (TLI ≥ .95, also known as the non-normed fit index or NNFI), and the root-mean-square error of approximation (RMSEA < .10) [35].

#### Differences by sex

We used one-way ANOVA to test for male vs. female differnces in the SBC-BA and SBC-BD mean scale scores. We compared two-factor models with both scales for males vs. females using an approach described by Byrne [36] to assess scale measurement properties by sex across samples, using four analytic steps. For each sample, we began the analyses by establishing separate measurement models for males and females. Then, moving though the four steps, we systematically compared different aspects of the two-factor measurement models for males vs. females. At each step in this process we compared the results with the prior step, examining for change in chi square per degrees of freedom and change in fit indices (AIC, BIC). When comparing the findings from one step with the previous step, a non-significant chi square difference test meant that the male and female measurement models were equivalent, that is they did not differ significantly from one another. The four steps in this analysis are summarized as follows:

Step 1—*Configural invariance*. We examined if the number of factors and the pattern of factor loadings for the SBC-BA and SBC-BD latent variables were equivalent for the male vs. female two factor measurement model.The indices of how well the data fit with this version of the model were used to assess the subsequent comparison Steps 2–4.
Step 2—*Factor loading invariance*. This step tested if the actual factor loadings were equivalent for male vs. female SBC-BA and SBC-BD latent variables. Then, we compared the results of Step 2 with Step 1, described above, using chi square difference tests and looking for any changes in fit indices (AIC/BIC).
Step 3—*Common residual covariance*. If no differences betweeen sexes were found, we proceeded with Step 3 to test for the equivalence of specific error (residual) covariances that were common in both the male and female measurement models. That is, any correlated error variances that were common to both male and female SBC measurement models were held as equivalent in the analysis.
Step 4—*Structural factor variance/covariance*. The final step tested for equivalence of the factor variances and covariances for male vs. female measurement models. This was done by constraining factor variances and covariances for males vs. females to be equal.

## Results

### Distribution properties and scale reliability

Table 2 summarizes distributional properties for the SBC scales. Mean values of the SBC-BA ranged from 2.23 to 2.74 and ranged from .95–1.41 for the SBC-DB, indicating overall moderate body awareness and relatively low bodily dissociation. Cronbach alpha coefficients for SBC-BA were acceptable [37, 38], ranging from .72 - .86. For SBC-BD, coefficients ranged from .63 - .81, were generally acceptable although the reliabilities for the Italian and Netherlands samples were low at .64, and .63, respectively.

**Table 2:** SBC scale means and distributional properties and internal consistency reliability.

Scale	Country	Mean ± SD	Skew	Kurtosis	Reliability Cronbach alpha
SBC-BA (12 items)	Italy	2.74 ± .57	-.42	.06	.82
	France	2.56 ± .63	-.48	.27	.83
	Netherlands	2.51 ± .38	.01	.04	.72
	Portugual	2.29 ± .75	-.24	-.22	.86
	USA (a)	2.36 ± .66	-.28	.08	.86
	USA (b)	2.23 ± .66	-.30	.35	.84
	USA (c)	–	–	–	–
	Israel	2.58 ± .66	-.31	-.19	.86
SBC-BD (8 items)	Italy	1.37 ± .52	.34	-.11	.64
	France	0.96 ± .63	1.36	2.98	.75
	Netherlands	1.41 ± .43	.01	.14	.63
	Portugual	0.95 ± .59	1.01	1.79	.71
	USA (a)	1.07 ± .61	.67	.60	.79
	USA (b)	0.94 ± .61	1.16	1.52	.81
	USA (c)	1.30 ± .73	.87	.91	.76
	Israel	–	–	–	–

Only the SBC-BD was administered in the USA (c) study; only the SBC-BA was administered in the Israeli study. Missing values indicated by dashes (–).

### Construct validity

Results from the final confirmatory factor analyses for all samples are detailed in Table 3. Most CFI values met standards for excellent fit (≥ .95) for the SBC-BA and SBC-BD models, and all were in the good range (≥ .90). Similarly with a few exceptions, TLI values met conventional standards, ranging from .86 - .99 across samples. RMSEA varied for SBC-BA models from .04 - .08 and SBC-BD models from .02–1.0, within the standard acceptable range. Overall, the findings showed a good fit between the proposed measurement models and the data, providing evidence of construct validity across samples. Structural correlations between the SBC-BA and SBC-BD latent factors were also calculated for each sample (Table 3, final column), values ranged from uncorrelated (0.03) to moderately correlated (-0.42). With the exception of the Portugal sample, all significant correlations were negative.

**Table 3:** CFA goodness of fit indices and SBC-BA/SBC-BD factor correlations by study sample.

SBC Scale	Country	CFI	TLI	RMSEA	SBC-BA/SBC-BD Factor Correlation
Body Awareness (12 items)	Italy	.95	.94	.05	-.42
	France	.90	.88	.08	-.37
	Netherlands	.92	.90	.05	-.30
	Portugual	.97	.95	.05	.24
	USA (a)	.97	.96	.04	.03
	USA (b)	.95	.93	.06	-.23
	USA (c)	–	–	–	–
	Israel	.96	.94	.06	–
Bodily Dissociation (8 items)	Italy	.96	.93	.05	
	France	.91	.86	.10	
	Netherlands	.94	.91	.05	
	Portugual	.97	.94	.06	
	USA (a)	.93	.89	.08	
	USA (b)	.99	.99	.02	
	USA (c)	.95	.92	.08	
	Israel	–	–	–	

Only the SBC-BD was administered in the USA (c) study; only The SBC-BA was administered in the Israeli study. Missing values indicated by dashes (–).

Across samples, item loadings on the SBC-BA and SBC-BD latent variables (Table 4) were statistically significant. For Body Awareness, the strongest loadings (i.e., items 12 and 14, range .52 - .78) focused on the integration of physical and emotional experience via attendance to and reflection on inner body awareness (e.g., “take cues from my body to help me understand how I feel” and “listen for information from my body about my emotional state”). This result is consistent with the original validation study.[4] The weakest SBC-BA loading was item 3 (“…notice my breathing becomes shallow when I am nervous,” range .20 - .49). For SBC-BD, the strongest loadings (i.e., items 11 and 20) reflected difficulty with expression of and attention to emotions. Item 16 on the SBC-BD scale (“distract myself from feelings of physical discomfort,” range .10 - .38) had the weakest loadings across samples.

**Table 4:** Confirmatory factor analysis: Item loadings for SBC scales.

SBC Scale Item Number & Description	Country							
**Body Awareness**	Italy	France	Netherlands	Portugual	USA (a)	USA(b)	USA (c)	Israel
1. Aware of tension	.46 (1.00)	.53 (1.00)	.48 (1.00)	.56 (1.00)	.50 (1.00)	.57 (1.00)	–	.59 (1.00)
3. Breathing shallow	.43 (1.45)	.33 (.99)	.20 (.57)	.46 (.96)	.42 (1.09)	.23 (.49)	–	.49 (1.08)
4. Notice response to touch	.39 (1.02)	.35 (.72)	.33 (.85)	.49 (.82)	.50 (1.01)	.33 (.68)	–	.52 (.86)
6. Notice body change when angry	.53 (1.62)	.41 (1.06)	.28 (.79)	.57 (1.30)	.59 (1.37)	.62 (1.31)	–	.63 (1.21)
8. Aware during sexual activity	.30 (.79)	.48 (1.20)	.21 (.52)	.48 (.86)	.44 (.97)	.39 (.79)	–	.37 (.60)
9. Can feel breath travel	.46 (1.63)	.51 (1.57)	.43 (1.34)	.51 (1.13)	.57 (1.43)	.39 (.90)	–	.53 (1.11)
12. Take cues from body	.62 (2.03)	.66 (1.66)	.52 (1.30)	.57 (1.17)	.65 (1.51)	.65 (1.25)	–	.52 (1.03)
13. Think about cause of discomfort	.52 (1.52)	.66 (1.55)	.52 (1.41)	.56 (1.10)	.48 (1.13)	.58 (1.12)	–	.69 (1.39)
14. Listen to body about emotional state	.72 (2.24)	.78 (1.87)	.54 (1.35)	.64 (1.34)	.69 (1.62)	.74 (1.52)	–	.71 (1.53)
15. Notice stress in body	.58 (1.54)	.60 (1.27)	.34 (.91)	.68 (1.29)	.47 (.98)	.80 (1.53)	–	.67 (1.41)
17. Note where tension is in body	.62 (2.12)	.64 (1.73)	.59 (1.86)	.64 (1.24)	.65 (1.51)	.68 (1.34)	–	.65 (1.35)
18. Notice peaceful experience	.55 (1.59)	.61 (1.66)	.43 (1.03)	.64 (1.28)	.57 (1.30)	.61 (1.27)	–	.27 (.46)
**Body Dissociation**	Italy	France	Netherlands	Portugual	USA (a)	USA(b)	USA (c)	Israel
2. Difficult to identify emotions	.69 (1.00)	.71 (1.00)	.41 (1.00)	.27 (1.00)	.65 (1.00)	.44 (1.00)	.65 (1.00)	–
5. Body feels frozen, numb	.18 (.36)	.19 (.33)	.28 (.73)	.28 (1.33)	.29 (.49)	.68 (1.73)	.57 (.95)	–
7. Looking at body from outside	.13 (.23)	.37 (.59)	.11 (.28)	.75 (2.84)	.44 (.70)	.54 (.82)	.42 (.58)	–
10. Feel separated from body	.30 (.46)	.57 (.76)	.15 (.41)	.82 (2.76)	.39 (.58)	.78 (1.54)	.50 (.67)	–
11. Hard to express emotions	.59 (.99)	.78 (1.21)	.76 (1.93)	.28 (1.24)	.75 (1.34)	.49 (1.02)	.65 (1.07)	–
16. Distract self from discomfort	.10 (.16)	.21 (.30)	.35 (.79)	.14 (.57)	.35 (.64)	.38 (.82)	.29 (.43)	–
19. Separated during sexual activity	.29 (.44)	.51 (.79)	.22 (.57)	.74 (3.01)	.43 (.65)	.68 (1.45)	N/A	–
20. Difficult to pay attention to emotions	.74 (1.13)	.75 (1.05)	.69 (1.90)	.34 (1.34)	.70 (1.18)	.50 (1.13)	.76 (1.11)	–

Reported are standardized coefficients with unstandardized coefficients in parentheses. Only the SBC-BD was administered in the USA (c) study; only the SBC-BA was administered in Israeli study. Missing values indicated by dashes (–).

Some item loadings showed little overall variation and some had considerable variation across samples. The SBC-BA items with the least variation in factor loadings (i.e., item 1, range .46 - .57 and item 17, range .59 - .68) focused on awareness of “tension” in the body. In contrast, the SBC-BA item with the most variation in factor loadings across samples (item 15, range .34 - .80) was specific to noticing “stress” in the body, possibly reflecting the more amorphosis quality of stress vs. the more distinctly noticable experience of tension. Overall, the SBC-BD factor loadings varied substantially across samples. However, there were some patterns of note. The factor loadings were more consistent for items focused on emotional disconnection (i.e., items 2, 11, 20) and least consistent for items focused on avoidance or sense of separation from the body (i.e., items 5, 7, 10, 16, 19). This is likely due to the similiarity in content of the emotional disconnection items (e.g., difficulty identifying, expressing or paying attention to emotions), whereas the avoidance items varied more in content (e.g., item 5 “my body feels frozen, as though numb, during uncomfortable situations” and item 16 “I distract myself from feelings of physical discomfort”).

### Differences related to participant sex

#### Mean responses

For SBC-BA, significant mean differences by sex were observed in four of the seven samples tested (p < .001). Except for the Netherlands sample, females generally reported higher levels of body awareness than males (see Table 5). Although statistically significant, the mean differences for SBC-BA were typically modest (less than .4 difference). For SBC-BD, only the Portugal sample showed mean differences by sex, with females endorsing higher bodily dissociation than males.

**Table 5:** F Tests for sex differences for SBC-BA and SBC-BD scales.

Country	Sex	SBC-BA	F test	SBC-BD	F test
		Mean (SD)	p value	Mean (SD)	p value
Italy	Female	2.83 (.53)	38.15, *p* < .001***	1.37 (.52)	0.01, *p* = .93
	Male	2.52 (.60)		1.37 (.50)	
France	Female	2.56 (.61)	0.11, *p* = .74	.97 (.64)	1.33, *p* = .25
	Male	2.61 (.84)		.79 (.44)	
Netherlands	Female	2.47 (.37)	1.28, *p* < .001***	1.43 (.43)	3.02, *p* = .08
	Male	2.64 (.41)		1.34 (.41)	
Portugal	Female	2.49 (.69)	68.38, *p* < .001***	1.01 (.62)	9.00, *p* = .003**
	Male	2.09 (.74)		.89 (.54)	
USA (a)	Female	2.40 (.62)	1.73, *p* = .19	1.11 (.63)	1.73, *p* = .19
	Male	2.30 (.72)		1.01 (.59)	
USA (c)	Female	–	–	1.28 (.71)	0.41, p = .52
	Male	–		1.34 (.83)	
Israel	Female	2.69 (.62)	29.23, *p* < .001***	–	–
	Male	2.40 (.69)		–	

** *p* < .01;

*** *p* < .001.

Only the SBC-BD was administered in the USA (c) study; only the SBC-BA was administered in the Israeli study. Missing values indicated by dashes (–). USA (b) sample did not include males, and thus not included in these analysis.

#### Model comparisons

Table 6 summarizes the findings from a series of male vs. female comparisons conducted, using confirmatory factor analysis, to assess the two-factor model by sex. In Step 1, we tested whether or not males vs. females differed with respect to the number of and pattern of factors in the respective measurement models. In Step 2, where the factor loadings for the two-factor SBC measurement model were assumed to be equal for males vs. females, we found no notable changes in model fit compared to findings in Step 1. This finding was also supported by only slight changes (≤ .01) observed in the model fit indicies [39]. In Step 3, which examined for differences in model common error covariances, there were no differences for the male vs. female measurement models. Thus, the findings indicated no differences in the measurement aspects of the model for males vs. females. In Step 4, chi-square difference tests were used to determine if the model factor structure, reflected in the covariances (correlations) and variances, were similar for males vs. females. In this step, in contrast to earlier steps, we found significant differences for all samples, except the Netherlands sample. That sample had very few male participants and thus it was difficult to obtain a reliable covariance estimate. Step 4 findings indicated that the correlation/covariance between the two latent factors, SBC-BA and SBC-BD, differed significantly for males vs. females in most samples. For instance, for males in the Portugese sample the correlation between SBC-BA and SBC-BD was .30; for females the correlation was .14. Likewise in the Italian sample, for males the correlation between SBC-BA and SBC-BD was—.33; for females the correlation was—.49. In summary, the overall tests for the equivalence of measurement model for males vs. females showed considerable consistency in the number and pattern of SBC factors and the factor loadings across the study samples. With repect to the structural aspect of the model, however, the relative strength of the associations (covariances/correlations) between the SBC-BA and SBC-BD latent factors tended to differ for males vs. females across the study samples.

**Table 6:** Tests for model equivalence by sex: Multi-group CFA across diverse community-based samples.

Model	Country N (males/females)	ML χ^2^	df	CFI	TLI	RMSEA	SRMR	χ^2^_diff_ ^c^	df_diff_ ^d^	P
**Model 1** Configural model *Test factor structure invariance—number of factors and factor—loadings across two groups*	Italy (180/396)	762.54	333	.82	.79	.07	.08	–	–	–
	Netherlands (321/103)	524.24	334	.83	.81	.05	.07	–	–	–
	Portugal (464/445)	1035.71	330	.86	.84	.07	.08	–	–	–
	USA (a) (119/162)	513.32	332	.88	.86	.06	.09	–	–	–
	USA (c) (59/233)	34.71	26	.98	.96	.05	.04	–	–	–
	Israel (231/377)	307.22	100	.92	.90	.08	.06	–	–	–
**Model 2** Factor loading Invariance *Test equivalence of factor loadings across two groups*	Italy	786.07	351	.81	.80	.07	.08	23.53	18	.17
	Netherlands	546.91	352	.83	.81	.05	.08	21.96	18	.23
	Portugal	1021.47	347	.87	.85	.07	.08	14.24	17	.65
	USA (a)	530.69	350	.88	.87	.06	.09	17.37	18	.50
	USA (c)	42.23	32	.97	.96	.05	.06	7.52	6	.28
	Israel	317.67	111	.92	.91	.08	.06	10.46	11	.49
**Model 3** Common Residual Variance *Simultaneously test equivalence of factor loadings and common residual variance across groups*	Italy	786.07	352	.82	.80	.07	.08	0.001	1	.97
	Netherlands^a^	NA	–	–	–	–	–	–	–	–
	Portugal	1023.59	351	.87	.86	.07	.08	2.12	4	.71
	USA (a)	531.02	352	.88	.87	.06	.09	0.33	2	.85
	USA (c)	42.36	33	.97	.97	.04	.06	0.14	1	.71
	Israel	321.78	114	.92	.91	.08	.06	4.11	3	.25
**Model 4** Invariance of Structural Model *Tests invariance of factor covariancs/variances*.	Italy	793.76	355	.81	.80	.07	.09	7.69	3	.05
	Netherlands	551.82	355	.82	.81	.05	.08	5.63	3	.13
	Portugal	1051.24	354	.86	.85	.07	.09	27.65	3	< .002
	USA (a)	541.05	355	.88	.87	.06	.10	10.03	3	.02
	USA (c) ^b^	NA	–	–	–	–	–	–	–	–
	Israel ^b^	NA	–	–	–	–	–	–	–	–

^a^ The Netherlands male and female samples had no common residual variances, thus not necessary to test (NA).

^b^ Only one of the two SBC scales were used in two studies; the analysis for structural covariance was not applicable (NA). That is, only the SBC-BD scale was administered in the USA (c) study; only the SBC-BA scale was administered in the Israeli study. The French and USA (b) samples were not included in these analyses because the datasets included either no males or an insufficient number of males for analyses.

^c^ χ^2^_diff_ = differences in chi square values between two models compared.

^d^ df_diff_ = difference in degrees of freedom between two models compared.

## Discussion

The primary study aims were to evaluate the psychometric properties, including the construct validity, of the SBC by examining data from multiple studies involving heterogeneous samples across many countries/languages. Overall, the results showed acceptable distributional properties and reliability coefficients, confirmed that the two SBC dimensions are not highly correlated and should be separately scored, and demonstrated SBC construct validity. We also examined for differences in mean scores by sex and assessed scale construct validity for males vs. females. While some of the included samples showed significant mean differences in male/female responses, the latent facture structure and factor loadings were stable across males and females in all of the measurement models. Overall, these results indicate that the SBC is a valid instrument for use in research involving community samples.

The CFA results show distinct differences in item loadings for the SBC-BA and the SBC-BD. The SBC-BA item loadings were stronger and more consistent across the study samples than the SBC-BD item loadings. Notably, the loadings of the SBC-BD items measuring emotional awareness were similar to the SBC-BA items in their strength and consistency. As found and noted in the original validation study[4], the strongest item loadings on *both* dimensions involved emotional awareness, indicating that emotional awareness is integral to both body awareness and bodily dissociation. Interdisciplinary and cognitive neuroscience models highlight the role of interoceptive awareness for emotion regulation [22], thus this observation may also point to fundamental links between body awareness, bodily dissocation and emotion regulation that are important for future study.

The results also revealed SBC mean differences by sex. Specifically, females vs. males reported signficantly higher body awareness in four of the six study samples, each from a distinct country/language. These findings suggest that females, as observed in the various study samples, tend to rate sensory awareness items higher than males, and thus may be more attuned to their bodies than males. In contrast, with the exception of the Portuguese sample, no sex differences were observed for the bodily dissociation scale, indicating that females and males are similar in their overall sense of disconnection from physical and emotional sensations. In the Portuguese sample, however, females had significantly higher bodily dissociation compared to males. The Portuguese sample is unique, compared to the other study samples, because the online recruitment strategy included a banner indicating that the survey was focused on sexual health. It is possible that recruitment specifically to a study on sexual health was linked with a self-selection bias in this sample in which sex differences in bodily dissociation might be more pronounced.

A number of study limitations need to be considered in interpreting the results. First, the majority of datasets were from countries with a germanic or latin language base; future research is needed to examine a broader range of samples by language/culture. Second, for more definitive comparisons, it would have been ideal to have multiple study samples from the same country and same language fluency. Third, the datasets based on translated scales typically involved only one study (with the exception of the Israeli dataset which combined data from multiple studies, which had used similar methods and samples). Fourth, mental health measures were not available for these studies, thus we were unable identify or exclude cases due to mental disorder. In addition, although the samples were large and the recruitment strategies were open to the general public, we do not know whether the samples are truly representative of the general populations. Last, not all datasets included demographic variables such as age, education, income or race, so we were unable to systematically address whether or not these factors influenced responses or factor structure.

Despite these limitations, this study offers a comprehensive and large scale validation of the SBC across multiple languages/countries focusing on community samples that were heterogeneous by age and sex. The results form a solid basis for the use of the SBC in research, and at the same time provides a validated comparison model for future studies with both community and clinical samples. The detailed and summarized findings of SBC-BA and SBC-BD responses for both males and females is likewise highly relevant for future comparisons of both community and clinical samples. In conclusion, the SBC is a brief, easy to administer, reliable and valid instrument for research involving the study of body awareness and bodily dissociation.

## Supporting information

S1 Scale and Scoring Information Appendix The Scale of Body Connection (SBC) and scoring instructions are in the S1 appendix. In addition the scale, scoring instructions, translations and other SBC information are available through the University of Washington Office of Nursing Research:https://nursing.uw.edu/research/research-tools/.
(RTF)
