# Supplementary material for: Scale of Body Connection: A multi-sample construct validation study
Source: PLoS One. 2017 Oct 13;12(10):e0184757. doi: 10.1371/journal.pone.0184757 (PMC5640211; doi:10.1371/journal.pone.0184757)
Supplement: S1 Scale and Scoring Information Appendix — In addition the scale, scoring instructions, translations and other SBC information are available through the University of Washington Office of Nursing Research:https://nursing.uw.edu/research/research-tools/. (RTF) [file pone.0184757.s001.rtf]

S B C
Study Number:  __________	Date:  ___________
Instructions:
This questionnaire asks about your body awareness and your response to body awareness.  
For each statement please check the box that best answers the way you generally feel.  
There are no right answers, please answer as truthfully as you can.
There are two questions about sexual activity; please consider all sexual activity including self-stimulation.  If you do not engage in sexual activity, please leave these questions blank.
	Not at all
0	A little bit
1	Some 
of the time
2	Most
 of the time
3	All of
 the time
4	
1.	If there is tension in my body, I am aware of the tension
						
2.	It is difficult for me to identify my emotions
						
3.	I notice that my breathing becomes shallow when I am nervous
						
4.	I notice my emotional response to caring touch 
						
5.	My body feels frozen, as though numb, during uncomfortable situations 						
6.	I notice how my body changes when I am angry
						
7.	I feel like I am looking at my body from outside of my body
						
8.	I am aware of internal sensation during sexual activity
						
9.	I can feel my breath travel through my body when I exhale deeply
						
10.	I feel separated from my body 
						
11.	It is hard for me to express certain emotions 
						
12.	I take cues from my body to help me understand how I feel
						
13.	When I am physically uncomfortable, I think about what might have caused the discomfort						
14.	I listen for information from my body about my emotional state 
						
15.	When I am stressed, I notice the stress in my body
						
16.	I distract myself from feelings of physical discomfort 
						
17.	When I am tense, I take note of where the tension is located in my body						
18.	I notice that my body feels different after a peaceful experience
						
19.  I feel separated from my body when I am engaged in 
  sexual activity						
20.  It is difficult for me to pay attention to my emotions
						
© Cynthia Price; cynthiap@uw.edu

Scale of Body Connection (SBC) Scoring 
Cynthia Price, PhD 

Box 357266 University of Washington, Seattle WA 98195
Email:cynthiap@uw.edu

There are 20 items on this scale, and two dimensions of Body Awareness and Bodily Dissociation.  The SBC should be scored as two separate scales of body awareness and body dissociation as these dimensions show little correlation to each other. 
Body awareness (BA) items  (12):  1,3,4,6,8,9,12,13,14,15,17,18
Body dissociation (DB) items (8):  2,5,7,10,11,16,19,20

Scoring  
The items are scored on a 5-point scale, ranging from 0-4 with 0 at “not at all” and 4 at “all of the time.” 

A positive change on the BA scale would represent an increase in bodily awareness, and a positive result.  A negative change on the DB scale would represent a decrease in bodily dissociation, and a positive result.

To score the BA subscale:  sum the endorsed items and divide by the total number of items (12).
To score the DB subscale: sum the endorsed items and divide by the total number of items (8).

Description of Measure:
The Scale of Body Connection (SBC) has two distinct, uncorrelated dimensions measuring body awareness and body association.  A 10-point scale, 12 items measure body awareness ( = .85) and 8 items to measure body association ( = .79).  Body awareness measures conscious attention to sensory cues indicating bodily state (for example tension, nervousness, peacefulness).  Body association measures connection or separation from body, including emotional connection (for example ease or difficulty attending to emotion).  The scale has demonstrated construct validity through exploratory and confirmatory factor analysis (Price and Thompson).

Reference:  Price, C. & Thompson, EA. (2007).  Measuring dimensions of body connection:  Body awareness and bodily dissociation.  Journal of Alternative and Complementary Medicine, 13(9):945-954.
